# Increased Inhibition May Contribute to Maintaining Normal Network Function in the Ventral Hippocampus of a Fmr1-Targeted Transgenic Rat Model of Fragile X Syndrome

**DOI:** 10.3390/brainsci13111598

**Published:** 2023-11-17

**Authors:** Leonidas J. Leontiadis, George Trompoukis, Panagiotis Felemegkas, Giota Tsotsokou, Athina Miliou, Costas Papatheodoropoulos

**Affiliations:** Laboratory of Neurophysiology, Department of Medicine, University of Patras, 26504 Rion, Greece; ljleontas@gmail.com (L.J.L.); geortrobjs@gmail.com (G.T. (George Trompoukis)); pfelemegkas@hotmail.gr (P.F.); panagiotatsotsokos@gmail.com (G.T. (Giota Tsotsokou)); athinamhliou@gmail.com (A.M.)

**Keywords:** fragile X, neurodevelopmental disorders, hippocampus, dorsoventral, septotemporal, excitation, inhibition, GABA_A_ receptors, epileptiform discharges, rat

## Abstract

A common neurobiological mechanism in several neurodevelopmental disorders, including fragile X syndrome (FXS), is alterations in the balance between excitation and inhibition in the brain. It is thought that in the hippocampus, as in other brain regions, FXS is associated with increased excitability and reduced inhibition. However, it is still not known whether these changes apply to both the dorsal and ventral hippocampus, which appear to be differently involved in neurodegenerative disorders. Using a Fmr1 knock-out (KO) rat model of FXS, we found increased neuronal excitability in both the dorsal and ventral KO hippocampus and increased excitatory synaptic transmission in the dorsal hippocampus. Interestingly, synaptic inhibition is significantly increased in the ventral but not the dorsal KO hippocampus. Furthermore, the ventral KO hippocampus displays increased expression of the α1GABA_A_ receptor subtype and a remarkably reduced rate of epileptiform discharges induced by magnesium-free medium. In contrast, the dorsal KO hippocampus displays an increased rate of epileptiform discharges and similar expression of α1GABA_A_ receptors compared with the dorsal WT hippocampus. Blockade of α5GABA_A_ receptors by L-655,708 did not affect epileptiform discharges in any genotype or hippocampal segment, and the expression of α5GABA_A_ receptors did not differ between WT and KO hippocampus. These results suggest that the increased excitability of the dorsal KO hippocampus contributes to its heightened tendency to epileptiform discharges, while the increased phasic inhibition in the Fmr1-KO ventral hippocampus may represent a homeostatic mechanism that compensates for the increased excitability reducing its vulnerability to epileptic activity.

## 1. Introduction

Fragile X syndrome (FXS) is a genetic, phenotypically complex disorder associated with several neuropsychological deficits, including hyperactivity, hypersensitivity, cognitive impairments, learning deficits, and sleep dysregulation, thereby representing a syndrome of intellectual disability [1,2,3,4,5,6]. Furthermore, approximately 30% of FXS patients are also diagnosed with autism, making FXS the most common inherited single-gene cause of autism [4,5,6]. The cause of FXS is the transcriptional silencing of the Fmr1 gene, leading to the suppression of expression of fragile X messenger ribonucleoprotein (FMRP) [7,8]. The widespread expression of FMRP in the brain makes it a key regulator of neuronal activity, and suppression of FMRP expression is associated with deficits in the function of neural circuits [9,10].

Neuronal hyperexcitability is a prominent neurobiological feature of FXS thought to result from a disturbed balance between excitation and inhibition (E-I) [11,12]. Accordingly, a consistent observation in the brains of patients and animal models of FXS is a reduction in GABA signaling, including GABAergic neurons [13], expression of GABA_A_ receptor subunits [14,15,16,17,18], GABA content [19,20], GABA release [21], GABA_A_ receptor-mediated synaptic currents [16,22], and dysfunctional inhibitory network [23,24,25,26]. However, not all studies agree with a monotonous brain-wide reduction of GABAergic inhibition in FXS [27,28].

Compatible with increased excitability and reduced inhibition, individuals with FXS display increased susceptibility to epilepsy [3,29], with epileptic seizures occurring frequently in young patients with FXS [30,31,32,33]. Interestingly, however, seizures occurring in children and teenagers with FXS usually disappear in adulthood [3,33,34,35], and seizures are rarely observed in adult patients.

Previously accumulated evidence shows that FXS-associated neurobiological changes are brain region-specific [36,37,38], and the hippocampus is among the brain regions that are affected by the loss of FMRP [39,40,41]. Nevertheless, the hippocampus is not a functionally homogeneous structure, displaying functional segregation along its long septotemporal or dorsoventral axis [42,43,44,45]. To a certain extent, the segregation of functions along the hippocampus could be attributed to specific anatomical connections of distinct segments of the hippocampus with extrahippocampal structures [45,46,47,48,49]. In addition, there is abundant evidence suggesting that large-scale functional segregation in the hippocampus may be accompanied by diversification of the intrinsic neuronal circuit along the long axis of the structure [42,50]. More specifically, recent evidence demonstrated that GABAergic inhibition is lower in the ventral compared with the dorsal segment of the hippocampus of adult rats [51,52,53,54,55]. The relatively reduced inhibition of the ventral hippocampus may contribute to the specific functional roles as well as characteristic susceptibility to epilepsy/epileptiform discharges of this segment of the hippocampus [56,57,58,59,60,61,62,63,64,65,66,67,68]. Notably, despite the evidence on the impact of FXS in the dorsal hippocampus, it is not yet known whether FXS similarly affects neuronal activity in the VH as well. Considering the above-described evidence, we wondered whether and how FXS affects synaptic transmission, neuronal excitability, and GABAergic inhibition in the dorsal and ventral hippocampus.

In the present study, we used a recently developed rat model of FXS, the Fmr1-knock-out (KO) rat model, and we examined basic features of the local neuronal network using slices from the dorsal hippocampus (DH) and the ventral hippocampus (VH) of adult rats in combination with field recordings of evoked potentials. We found that neuronal excitability is enhanced in both DH and VH of Fmr1-KO rats. Interestingly, in the VH, but not the DH, the increase in excitability is accompanied by an increase in the effectiveness of inhibition and upregulation of α1 subunit-containing GABA_A_ receptors but not α5 subunit-containing GABA_A_ receptors. Furthermore, the VH of Fmr1-KO rats displays a striking resistance to induced epileptiform activity, while the KO DH displays increased epileptiform activity. Our results show that the DH and VH respond unequally to the loss of FMRP, suggesting that FXS may be associated with distinct localized alterations even inside a particular brain structure; furthermore, our data point to the possibility that some changes occurring in the brain of subjects suffering from neurodevelopmental disorders may represent homeostatic processes that attempt to maintain the effectiveness of the neuronal network function.

## 2. Materials and Methods

### 2.1. Animals and Hippocampal Slices

In this study, we used adult male Long Evans rats, 3–4 months old. Both wild-type (WT) and Fmr1-KO (KO) rats were obtained from the Medical College of Wisconsin (RRIDs: RGD_ 2308852 and RGD_ 11553873, respectively). Rats were maintained at the specific pathogen-free Laboratory of Experimental Animals of the Department of Medicine of the University of Patras (license No: EL-13-BIOexp-04). Rats were kept under a stable cycle of light–dark (12/12 h) and a temperature of 20–22 °C; animals had free access to food and water. The treatment of rats and all experimental procedures we used were conducted in accordance with the European Communities Council Directive Guidelines for the care and use of Laboratory animals (2010/63/EU—European Commission). Furthermore, they were approved by the Protocol Evaluation Committee of the Department of Medicine of the University of Patras and the Directorate of Veterinary Services of the Achaia Prefecture of Western Greece Region (reg. number: 5661/37, 18 January 2021), and this animal study was reviewed and approved by the Research Ethics Committee of the University of Patras. Rats were genotyped after each experiment using tail or brain tissue to test the expression of FMRP protein by means of Western blotting.

Slices were prepared from the dorsal and the ventral segment of the hippocampus as previously described [69]. Briefly, after decapitation of the animal under anesthesia with diethyl-ether and removal of the brain from the skull, we sliced the dorsal and ventral hippocampus transversally to its long axis using a McIlwain tissue chopper. Specifically, we prepared 500 μm thick slices between 0.5 mm and 3.5 mm from the dorsal and ventral end of the hippocampus. We performed the surgical procedure with the tissue submerged in chilled (2–4 °C) artificial cerebrospinal fluid (aCSF) containing (in mM) 124 NaCl, 4 KCl, 2 CaCl_2_, 2 MgSO_4_, 26 NaHCO_3_, 1.25 NaH_2_PO_4_, and 10 glucose, and equilibrated with 95% O_2_ and 5% CO_2_ gas mixture at a pH = 7.4. We immediately transferred slices in a homemade interface-type recording chamber where they were continuously perfused with aCSF of the same composition as described above at a temperature of 30 ± 0.5 °C.

### 2.2. Electrophysiological Recordings

Recordings were started at least one and a half hours after the placement of the slices in the chamber. We recorded evoked field excitatory synaptic potentials (fEPSPs) and population spikes (PSs) from the stratum radiatum and stratum pyramidale, respectively, of the CA1 hippocampal region, using a 7 μm-thick carbon fiber electrode (Kation Scientific, Minneapolis, MN, USA). Field potentials were evoked following electrical stimulation of Schaffer collaterals using a homemade bipolar platinum/iridium wire electrode with a wire diameter of 25 μm (World Precision Instruments, Sarasota, FL, USA) and an inter-wire distance of 100 μm. Electrical stimulation consisted of constant current pulses with a stable duration of 100 μs and variable amplitude (20–300 μA). We applied baseline stimulation at a frequency of 0.033 Hz using a current stimulation strength, eliciting an fEPSP with a slope of approximately 1 mV/ms or a PS with an amplitude of roughly 1 mV. We quantified fEPSP by the maximum slope of the early rising phase and PS by its amplitude measured as the length of the projection of the minimum peak on the line connecting the two maxima peaks of the PS waveform. From input–output curves constructed between the stimulation current intensity and fEPSP or PS, we assessed synaptic effectiveness and neuronal excitation, respectively. We also assessed neuronal excitability by the relationship between fEPSP and PS (i.e., the PS/fEPSP ratio). We studied the effectiveness of feedback inhibition in suppressing principal cell firing in the local neuronal circuit using a paired-pulse stimulation protocol. Specifically, we delivered, in rapid succession (10 ms), two pulses of identical intensity and duration at the Schaffer collaterals, and we estimated the so-produced paired-pulse inhibition (PPI) by measuring the suppression of PS evoked by the second pulse with respect to PS evoked by the first pulse.

Spontaneous population discharges resembling interictal epileptiform discharges were induced by removing magnesium ions (Mg^2+^) from the perfusion medium (i.e., Mg^2+^-free medium). The effects of an inverse agonist of α5GABAARs L-655,708 (Tocris Cookson Ltd., Bristol, UK) were examined in epileptiform population discharges. Epileptiform discharges were quantified by the probability of their appearance in slices from individual rats and their frequency (rate) of occurrence in individual hippocampal slices. Measures of epileptiform discharges were obtained after approximately ninety minutes of tissue perfusion with Mg^2+^-free medium when a stable rate of discharge occurrence was established. The electrophysiological signal was acquired and amplified X500 and then filtered at 0.5 Hz–2 kHz using Neurolog amplifiers (Digitimer Limited, Welwyn Garden City, UK); the signal was digitized at 10 kHz and stored on a computer disk for offline analysis using the CED 1401-plus interface and the Spike2 version 5 software, respectively (Cambridge Electronic Design, Cambridge, UK).

### 2.3. Western Blotting

The CA1 region of the dorsal and ventral hippocampus from WT and KO rats and the remaining brain tissue or tail tissue were stored at −80 °C for protein expression analysis. Tissue was solubilized in 200 μL lysis buffer containing 1% SDS and protease inhibitors at a 1:100 dilution and homogenized with sonication. Protein concentration was determined for each sample using the NanoDropTM 2000/2000c Spectrophotometer. Protein homogenates (25–50 μg of protein per lane) were subjected to sodium dodecyl sulfate-polyacrylamide gel electrophoresis (SDS-PAGE) on 10% polyacrylamide gels for 30 min at 80 V and 1 h at 120 V. Proteins were transferred to polyvinylidene difluoride (PVDF) membrane at 400 mA for 90 min followed by 1 h of blocking at room temperature (RT) in PBS containing 0.1% Tween-20 (PBST) and 5% nonfat dried milk. Membranes were next incubated overnight at 4 °C with the following primary antibodies diluted in PBST, 3% dried milk: rabbit anti-FMRP polyclonal (1:1500 dilution, #17722, Abcam, Cambridge, UK), rabbit anti-GABAA α1 R polyclonal (1:2500, #06-868, Millipore Sigma, Burlington, MA, USA), mouse anti-GABAA α5 R monoclonal (1:1000, #ΜA5-27700, Thermo Fisher Scientific, Waltham, MA, USA), and rabbit anti-β-actin polyclonal (1:15,000, #E-AB-20058, Elabscience, Houston, TX, USA) antibodies. The blots were rinsed with PBST and then incubated with either goat anti-rabbit or anti-mouse secondary horseradish peroxidase-conjugated IgG antibodies for 60 min at RT. Immunodetection was carried out using an Enhanced Chemiluminescence detection system. The bands were visualized on ChemiDoc MP (BioRad, Hercules, CA, USA) with 1 to 10 min exposures. Optical density measurements from each band were defined as ROD units with ImageLab 6.1. The ROD of each band was quantified relative to the ROD of β-actin, which serves as a gel-loading control. Then, the ratio (ROD of protein of interest)/(ROD β-actin) was normalized with the same ratio of an internal sample, which was loaded in all gels.

### 2.4. Statistics

The parametric independent *t*-test, paired *t*-test, and the two-way ANOVA were used to assess the effects of genotype, hippocampal segment, or drug on the various parameters. Whenever variances differed between compared populations of values, we used parametric tests that accounted for unequal variances. We performed statistical analysis on electrophysiological data using the number of slices. However, Western blot data were analyzed using the number of rats. The IBM SPSS Statistics 27 software package was used for all statistical analyses. Values throughout the text represent mean ± S.E.M.

## 3. Results

### 3.1. Synaptic Transmission and Neuronal Excitability

**Figure 1:**
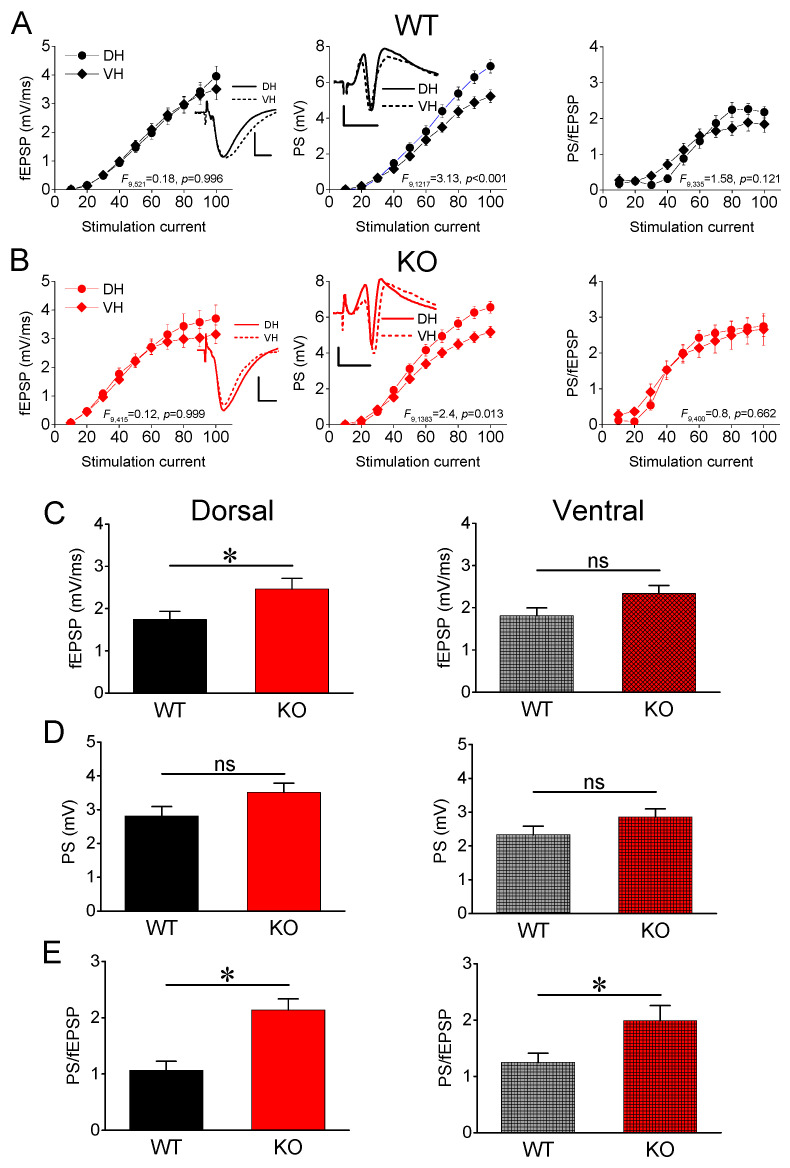
(**A**,**B**) Input–output curves of fEPSP, PS, and PS/fEPSP as a function of stimulation current intensity in DH and VH of WT (**A**) and KO rats (**B**). At the bottom of graphs A and B are shown the results of statistical analysis (two-way ANOVA) of input–output curves (effect of the interaction between hippocampal segment and stimulation current intensity). Example traces of fEPSP and PS are shown in inserts; calibration bars: 1 mV, 5 ms. (**C**–**E**) Effects of genotype on fEPSP (**C**), PS (**D**), and PS/fEPSP (**E**) in DH (left panel) and VH (right panel). Average values of the three variables produced by stimulation current intensity of 40–70 μA are shown. Asterisks denote statistically significant difference between WT and KO (independent *t*-test). “ns” denotes not significant.

After constructing input–output curves between stimulation current intensity and evoked responses (fEPSP, PS and PS/fEPSP), we calculated the average fEPSP, PS, and PS/fEPSP produced by moderate stimulation current intensity (current intensity of 40–70 μA). First, we compared responses between the two segments of the hippocampus in WT rats. In keeping with previous results [53,69,70,71,72,73], we found that the average fEPSP recorded from WT rats did not significantly differ between the DH (n = 24) and VH hippocampus (n = 24) (independent *t*-test, t_46_ = −0.244, *p* = 0.808). Regarding the excitation of local neuronal circuitry, we found that the average PS was similar in DH-WT (n = 66) and VH-WT (n = 52) (independent *t*-test, t_116_ = 1.262, *p* = 0.209). We assessed the excitability of the local neuronal network by measuring the PS/fEPSP ratio and found no significant difference between DH-WT (n = 24) and VH-WT (n = 19) (independent *t*-test, t_32_ = −0.8, *p* = 0.430). These results are in accordance with previous evidence [72,74]. We obtained similar results when we compared DH-KO and VH-KO. Specifically, fEPSP (independent *t*-test, t_36_ = 0.4, *p* = 0.692), PS (independent *t*-test, t_135_ = 1.8, *p* = 0.074), and PS/fEPSP (independent *t*-test, t_36_ = 0.446, *p* = 0.658) were similar between DH-KO (n = 19, n = 72 and n = 19 for fEPSP, PS and PS/fEPSP) and VH-KO (n = 19, n = 65 and n = 19 for fEPSP, PS, and PS/fEPSP). We also explored the effect of the hippocampal segment on input–output curves in both genotypes (Figure 1A,B). We found similar results to those yielded by the *t*-test for synaptic transmission (fEPSP, WT: *F*_521_ = 0.25, *p* = 0.62; KO: *F*_415_ = 0.573, *p* = 0.449) and excitability (PS/fEPSP, WT: *F*_335_ = 1.24, *p* = 0.267; KO: *F*_400_ = 1.94, *p* = 0.165); however, we found increased excitation (PS) in the DH compared with VH in both WT (*F*_1217_ = 36.92, *p* < 0.001) and KO rats (KO: *F*_1383_ = 32.86, *p* < 0.001). We obtained similar results when we explored the interaction of hippocampal segment and stimulation current intensity on input–output curves, which are provided in the graphs of Figure 1A,B.

Then, we examined possible differences in fEPSP, PS, and PS/fEPSP between WT and KO rats (Figure 1C–E). Regarding excitatory synaptic transmission, we found that the genotype significantly affected fEPSP in DH but not VH (Figure 1C). Specifically, DH-KO (n = 19) displayed a significantly increased fEPSP compared with DH-WT (n = 24) (independent *t*-test, t_41_ = −2.314, *p* = 0.026) (Figure 1C, Dorsal). In contrast, the fEPSP recorded from VH did not significantly differ between WT (n = 24) and KO rats (n = 19) (independent *t*-test, t_41_ = −1.96, *p* = 0.057) (Figure 1C, Ventral). Furthermore, we found that the genotype did not significantly affect PS in either segment of the hippocampus (Figure 1D). Specifically, we found a similar PS between DH-WT (n = 66) and DH-KO (n = 72) (independent *t*-test, t_136_ = −1.79, *p* = 0.076) and between VH-WT (n = 52) and VH-KO (n = 65) (independent *t*-test, t_115_ = −1.543, *p* = 0.126). The neuronal excitability, however, assessed by the PS/fEPSP ratio significantly increased in both hippocampal segments of KO compared with WT rats (Figure 1E). Specifically, we found a significantly higher PS/fEPSP ratio both in DH-KO (n = 19) compared with DH-WT (n = 16) (independent *t*-test, t_33_ = −4.153, *p* < 0.001) and in VH-KO (n = 19) compared with VH-WT (n = 18) (independent *t*-test, t_35_ = −2.358, *p* = 0.024). These results indicate that excitatory synaptic transmission increases in DH-KO but not VH-KO, while neuronal excitability increases in both segments of the hippocampus in KO vs. WT rats.

### 3.2. Paired-Pulse Inhibition (PPI)

**Figure 2:**
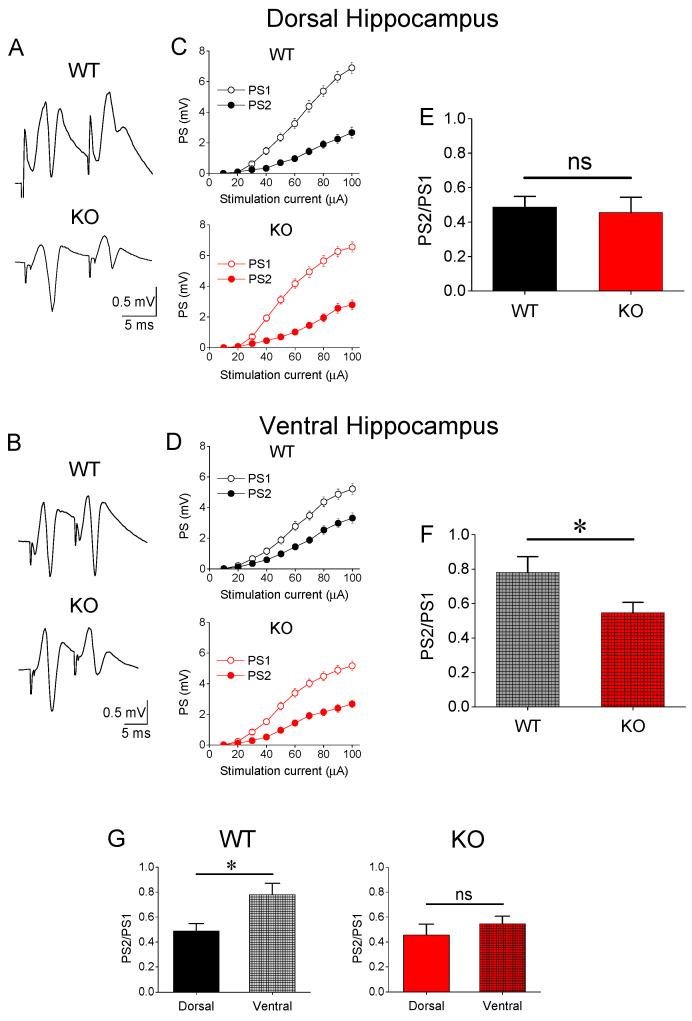
Paired-pulse inhibition is enhanced in VH-KO but not DH-KO. (**A**,**B**) Examples of trace recordings of the conditioning PS (PS1) and the conditioned PS (PS2) evoked by the paired-pulse stimulation in DH and VH, respectively, obtained from WT and KO rats. (**C**,**D**) Examples of input–output curves of PS1 and PS2 plotted as a function of stimulation current. Note that PS2 is suppressed more in DH-WT than in VH-WT, and that the suppression of PS2 is stronger in VH-KO than in VH-WT. (**E**,**F**) Collective data from DH and VH, respectively, showing that the average PS2/PS1 ratio is significantly lower in the VH-KO compared with VH-WT but similar in DH-WT and DH-KO. (**G**) Rearranged data to illustrate that the significant difference in PPI between DH-WT and VH-WT is eliminated in KO rats. Asterisks denote a statistically significant difference at *p* < 0.05 (independent *t*-test). Error bars represent SEM. “ns” denotes statistically not significant.

We examined PPI in DH and VH of WT and KO rats (Figure 2A–F). As previously demonstrated [51,52,53,54,55], we found that DH from WT rats displayed a significantly lower PS2/PS1 ratio compared with VH (independent *t*-test, t_115_ = −2.742, DH = 65 and VH = 52, *p* = 0.007). Then, the PS2/PS1 ratio observed in DH and VH was compared between WT and KO. We found that genotype did not significantly affect PS2/PS1 in the DH-KO (n = 65) compared with DH-WT (n = 72) (independent *t*-test, t_135_ = 0.294, *p* = 0.769) (Figure 2E). Remarkably, however, we found a significantly enhanced PPI in the VH of KO compared with WT rats. Specifically, we found a significant reduction in the PS2/PS1 ratio of VH-KO (n = 65) vs. VH-WT (n = 62) (independent *t*-test, t_115_ = 2.207, *p* = 0.029) (Figure 2F). Markedly, the increase in inhibition that occurred in the ventral hippocampus of KO rats led to the abolition of the inhibition difference between DH-WT (n = 72) and VH-WT (n = 65) (independent *t*-test, t_135_ = −0.833, *p* = 0.406) (Figure 2G). These results demonstrated that Fmr1-KO is associated with an enhancement of feedback inhibition in the CA1 field of the VH but not the DH.

### 3.3. Expression of α1 GABA_A_ Receptors

**Figure 3:**
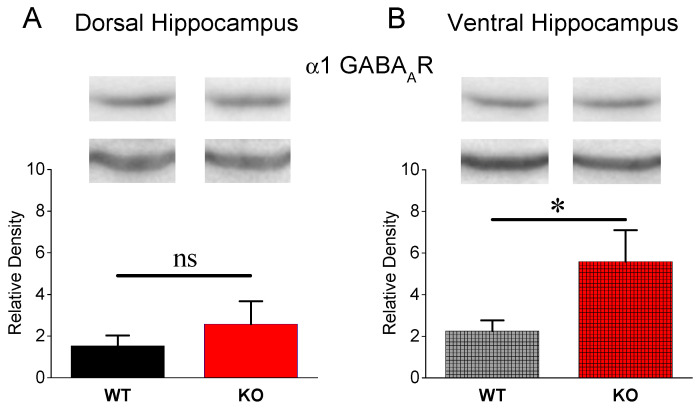
The protein expression of α1GABA_A_R is similar in DH-WT and DH-KO (**A**) but higher in VH-KO compared with VH-WT (**B**). Asterisks denote a statistically significant difference at *p* < 0.05 (independent *t*-test). “ns” denotes statistically not significant.

The increase in PPI found in the VH of KO vs. WT rats prompted us to further define whether the electrophysiological evidence is accompanied by a similar change at the molecular level. Therefore, we examined the protein expression of GABA_A_ receptors containing the α1 subunit (α1GABA_A_Rs), which present a dominant expression in the CA1 hippocampal field [75] and are mostly located at synaptic sites [76]. Figure 3 shows that a1GABA_A_Rs are similarly expressed in DH-WT (n = 8 rats) and DH-KO (n = 8 rats) (independent *t*-test, t_14_ = −0.865, *p* = 0.408). In contrast, we found a significantly higher expression of α1GABA_A_Rs in VH-KO (n = 10 rats) compared with VH-WT (n = 10 rats) (independent *t*-test, t_18_ = −2.1, *p* = 0.049). These results clearly corroborated the enhanced effectiveness of phasic feedback inhibition in the VH-KO.

### 3.4. Epileptiform Activity

**Figure 4:**
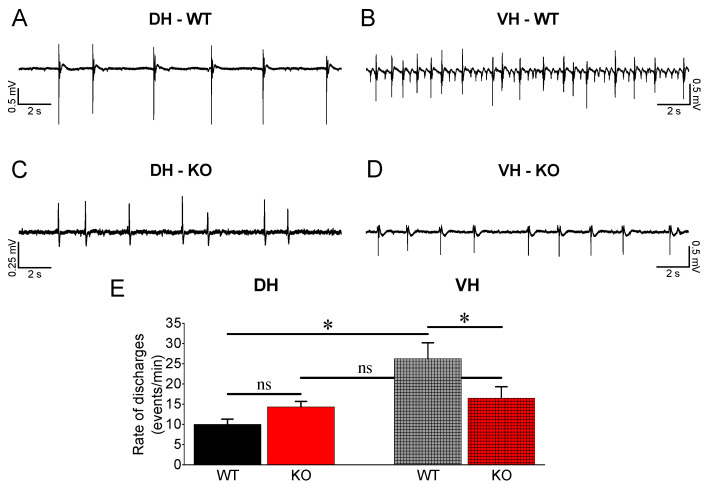
Comparison of Mg^2+^-free-induced population discharges between WT and KO. (**A**–**D**) Example trace recordings from DH (left panel) and VH (right panel) of WT and KO rats (**E**). Collective data are shown. Asterisks denote a statistically significant difference at *p* < 0.05 (independent *t*-test). “ns” denotes statistically not significant. Note that epileptiform discharges occur less frequently in DH-WT than VH-WT; they occur with similar frequency in DH-WT and DH-KO, but their frequency is reduced in VH-KO compared with VH-WT.

Considering that the ventral hippocampus in rodents and the corresponding anterior hippocampus in humans display increased susceptibility to epileptic/epileptiform activity compared with the dorsal hippocampus [56,62,64,66,68,77,78,79] and that the relatively reduced inhibition in the ventral compared with dorsal hippocampus [51,52,53] may significantly contribute to this susceptibility, we wondered whether the increase in feedback inhibition in VH-KO observed here could have an effect on the vulnerability of this hippocampal segment to epileptiform activity. Thus, we induced spontaneous epileptiform discharges in DH and VH from WT and KO rats perfusing slices with medium without magnesium ions (Mg^2+^-free medium). Under these conditions, we observed interictal-like population discharges in both DH and VH from WT and KO rats (Figure 4).

Epileptiform discharges appeared with increased incidence in VH-WT (n = 54) compared with DH-WT (n = 67) (independent *t*-test, t_63.08_ = −3.954, *p* < 0.001) (Figure 4Ε) as previously described [62,64,67,68]. Comparing the rate of discharges between DH-WT (n = 67) and DH-KO (n = 58), we found no significant difference (independent *t*-test, t_120_ = −2.18, *p* = 0.05). In contrast, the rate of discharges was significantly lower in VH-KO (n = 51) compared with VH-WT (n = 54) (independent *t*-test, t_94.68_ = 2.01, *p* = 0.047). As a result, we found no difference in the rate of discharges between DH and VH in the KO rats (independent *t*-test, t_72.61_ = −0.713, *p* = 0.478). These results led us to conclude that the enhancement of PPI accompanied by an upregulation of α1GABA_A_ receptors effectively contributes to reducing the rate of epileptiform discharges in the VH of KO rats.

### 3.5. Effect of SR 95531 on Epileptiform Population Discharges

**Figure 5:**
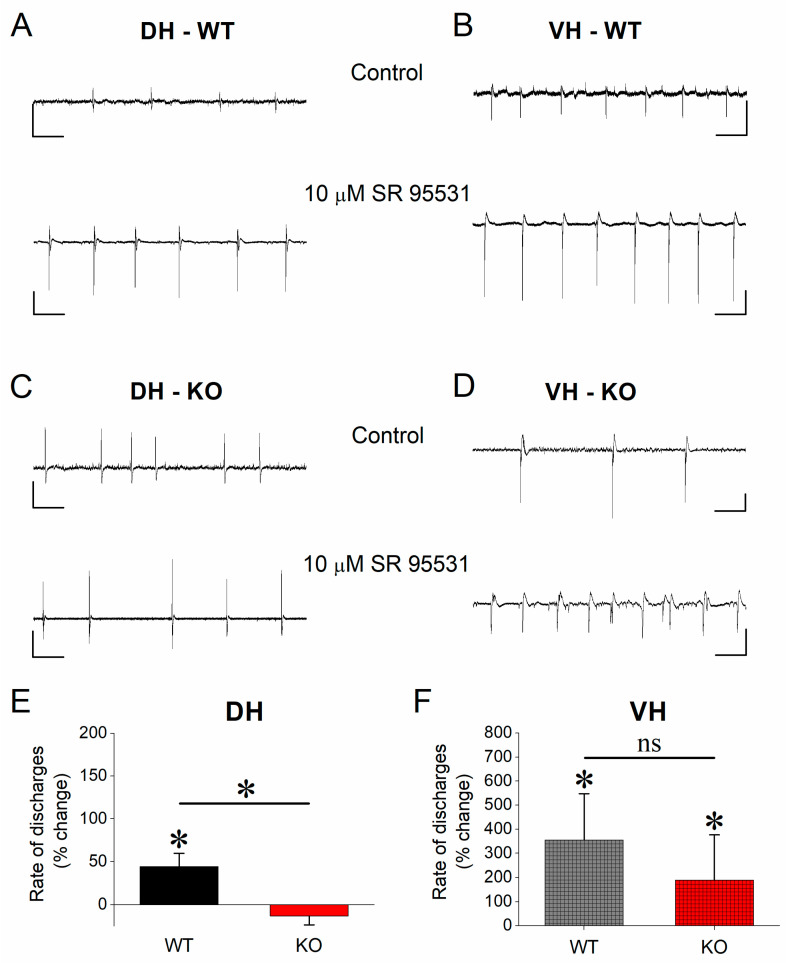
Effects of SR 95531 on epileptiform population discharges. (**A**–**D**) Example trace recordings from DH and VH of WT and KO rats, obtained under control conditions and during application of SR 95531. Calibration bars: 0.5 mV, 2 ms. (**E**,**F**) Collective data are shown for DH (**E**) and VH (**F**). Asterisks denote a statistically significant difference at *p* < 0.05 (independent *t*-test). “ns” denotes statistically not significant.

Assuming the increased inhibition contributes to the reduction of the vulnerability of KO vs. WT VH to epileptiform activity, we hypothesized that suppression of inhibition should eliminate the genotype-related difference in the rate of epileptiform population discharges in this segment of the hippocampus. First, considering that α1GABA_A_Rs are located predominately at synaptic sites mediating phasic inhibition [76], we used the antagonist of GABA_A_ receptors SR 95531, which blocks phasic but not tonic inhibition in CA1 hippocampal neurons [80,81]. We applied SR 95531 to hippocampal slices perfused with a Mg^2+^-free medium (control condition). We observed that SR 95531 significantly increased the rate of epileptiform discharges in DH-WT (paired *t*-test, t_12_ = −2.28, *p* < 0.05, n = 13) but not in DH-KO (paired *t*-test, t_11_ = 1.59, *p* = 0.141, n = 12) (Figure 5A,C,E). Furthermore, application of SR 95531 significantly increased the rate of discharges in both VH-WT (paired *t*-test, t_5_ = −2.56, *p* = 0.049, n = 6) and VH-KO (paired *t*-test, t_7_ = −3.7, *p* = 0.018, n = 8) (Figure 5B,D,F), eliminating the difference in the rate of discharges in VH between WT and KO rats (independent *t*-test, t_24_ = 0.826, *p* = 0.417, n = 9 and n = 17 for WT and KO, respectively). Paradoxically, the rate of discharges was reduced in DH-KO compared with DH-WT under the action of SR 95531 (independent *t*-test, t_23_ = 3.042, *p* = 0.006, n = 16 and n = 21 for WT and KO, respectively).

### 3.6. Effect of L-655,708 on Epileptiform Population Discharges

**Figure 6:**
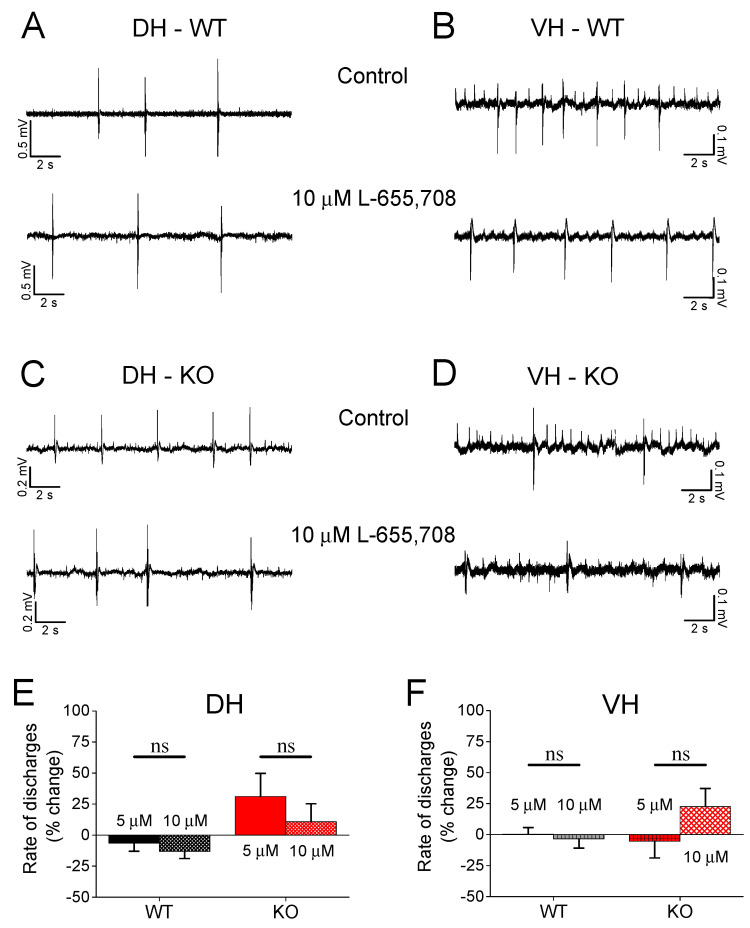
Effects of L-655,708 on epileptiform population discharges. (**A**–**D**) Example trace recordings from DH and VH of WT and KO rats, obtained under control conditions and during application of L-655,708. (**E**,**F**) Collective data are shown for DH (**E**) and VH (**F**). “ns” denotes statistically not significant. L-655,708 does not significantly affect the rate of epileptiform discharges in either segment of the hippocampus or genotype.

The previous experiment showed that the SR 95531 eliminates the difference in the rate of population discharges between VH-WT and VH-KO, suggesting that GABA_A_ receptor-mediated phasic inhibition plays a significant role in limiting the susceptibility of the VH-KO to epileptiform activity. However, GABA_A_ receptors also mediate tonic inhibition when located at extrasynaptic sites [82]. Therefore, the previous results with SR 95531, which blocks synaptic inhibition, could not apparently provide an answer to whether tonic inhibition may also play a role in the reduced rate of epileptiform discharges observed in VH-KO. Thus, we aimed to explore the possible involvement of tonic inhibition in the reduced susceptibility of the VH-KO to epileptiform activity, focusing on the α5 subunit containing GABA_A_Rs (α5GABA_A_Rs), which are largely extrasynaptic [76], greatly contribute to tonic inhibition [83,84], and are abundantly expressed in the hippocampus [85]. We used L-655,708, an inverse agonist of α5 subunit containing GABA_A_Rs (α5GABA_A_Rs), which suppresses tonic inhibition [83,86]. We applied L-655,708 at the concentration of 5 μM and 10 μM in hippocampal slices, which displayed epileptiform discharges in Mg-free medium. We observed that L-655,708 did not significantly affect the rate of epileptiform discharges in either hippocampal segment or genotype (Figure 6). Specifically, we found a similar rate of discharges before and after drug application in DH-WT (5 μΜ, independent *t*-test, n = 17, t_16_ = 1.9, *p =* 0.076; 10 μΜ, t_16_ = 3.2, *p =* 0.05) and DH-KO (5 μΜ, n = 12, t_8_ = −2.0, *p =* 0.07; 10 μΜ, t_8_ = 0.037, *p =* 0.97). Similarly, L-655,708 did not significantly affect discharges in VH-WT (5 μΜ, n = 12, t_11_ = −0.247, *p =* 0.809; 10 μΜ, t_11_ = 0.871, *p =* 0.402) and VH-KO (5 μΜ, n = 13, t_12_ = −1.02, *p =* 0.328; 10 μΜ, t_12_ = −2.08, *p =* 0.06).

### 3.7. Normal Protein Expression of α5 GABA_A_ Receptors in KO Dorsal and Ventral Hippocampus

**Figure 7:**
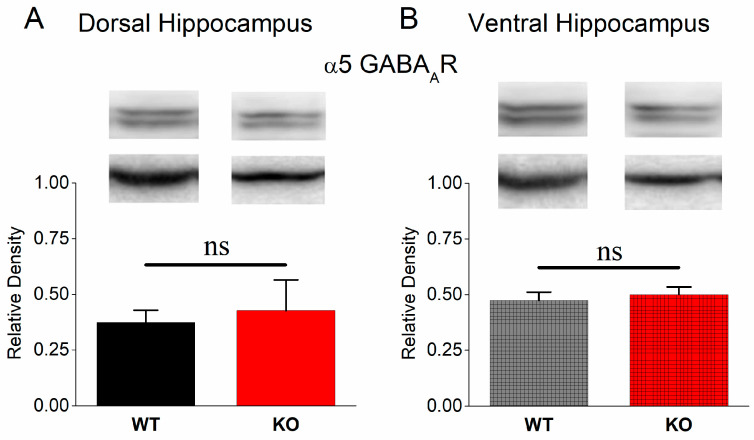
Protein expression of α5GABA_A_R in DH (**A**) and VH (**B**) from WT and KO rats. α5GABA_A_R displays similar expression between WT and KO hippocampus. “ns” denotes statistically not significant.

Our next aim was to confirm the above-described electrophysiological results at the molecular level. As shown in Figure 7, α5GABA_A_Rs display a similar expression in the DH (independent *t*-test, n = 5 WT and 5 KO rats, t_8_ = −0.358, *p =* 0.73) and VH (independent *t*-test, n = 5 WT rats and n = 4 KO rats, t_7_ = −0.506, *p =* 0.63) of WT and KO rats. This data set showed that α5GABA_A_Rs did not significantly change in KO rats and suggested that α5GABA_A_R-mediated tonic inhibition does not significantly participate in shaping the properties of epileptiform activity either in the DH or the VH. Alternatively, the absence of the effect of L-655,708 might also be related to the developmental reduction of tonic GABAergic current and α5GABAAR expression, which stabilize at a low level before adulthood [87].

## 4. Discussion

This study shows altered excitatory and inhibitory synaptic transmission and neuronal excitability in the hippocampus of Fmr1-KO adult rats. However, these changes are not equally expressed in the two segments of the hippocampus. Interestingly, the effectiveness of inhibition in limiting neuronal excitation is enhanced in the VH-KO vs. VH-WT but remains unaltered in DH-KO compared with DH-WT. The increased inhibition in VH-KO is associated with an enhanced expression of α1GABA_A_Rs and a notable restrain of induced epileptiform activity. These data suggest that a possible reorganization of the local neuronal network attempts to keep adult VH-KO functional, away from a state of hyperexcitability that could disrupt information processing.

A main neurophysiological correlate of FXS is an alteration of E-I balance towards excitation [11,12]. In keeping with previous observations [88,89,90,91,92,93,94], we report that loss of FMRP is accompanied by increased excitability of the hippocampal network. Additionally, it is widely thought that a crucial determining factor in the FXS-associated increase of the E-I ratio is the reduction in inhibition [95,96]. Indeed, GABAergic inhibition has been extensively studied in the KO cortex, and FMRP modulates the function of GABA_A_ receptors in the hippocampus [97]. However, studies of GABAergic inhibition in the hippocampus are relatively few [13,16,21,22,98]. We found three studies that examined GABAergic synaptic transmission in the CA1 hippocampal field [16,21,98]. All these studies have been performed in young or immature animals, particularly in the dorsal or medial segment of the mouse hippocampus. Sabanov and collaborators reported reduced expression in α2, β1, and δ GABA_A_ receptor subunits and reduced phasic and tonic inhibitory currents in CA1 pyramidal cells. The other two studies found defective presynaptic GABA_B_ receptor-mediated signaling at Schaffer collaterals [21,98]. Additionally, previous studies have shown that the absence of FMRP is accompanied by selective changes in the expression of GABA_A_ receptor subunits in the adult hippocampus, which displays decreased expression of β2, increased expression of β3, and no change in expression of β1 subunits [18,99]. Additionally, decreased surface expression of the δ GABA_A_ receptor subunit has been recently observed in the hippocampus [22]. Nevertheless, these studies do not account for possible dorsoventral differences in GABAergic inhibition in FXS. The present is the first study that comparatively examines basal electrophysiological phenomena in the dorsal and ventral hippocampus of Fmr1-KO rats. An increase in GABAergic inhibitory actions, such as that found here, should reasonably be accompanied by an upregulation of GABA_A_R subtypes that mediate a relatively increased postsynaptic effect, especially α1GABA_A_Rs that permit an increased hyperpolarizing current [100]. Thus, the increased expression of the α1 subunit of GABA_A_Rs in the ventral KO hippocampus suggests that the enhanced inhibition may result from an upregulation of α1GABA_A_R in the ventral KO hippocampus. Notably, in contrast to the α1 subunit, we found that the α5 subunit is normally expressed in VH-KO. Considering that α1 and α5 subunits predominately participate in synaptic and extrasynaptic GABA_A_Rs, respectively [76], these data suggest that an increase in phasic but not tonic GABA_A_R-mediated transmission occurs in VH of KO vs. WT adult rats. The absence of change in inhibition in the dorsal hippocampus of adult KOs is consistent with previous observations showing a stable number of GABAergic neurons in this segment of the hippocampus between WT and Fmr1-KO adult mice [13] and is compatible with the lack of change in phasic GABAergic inhibition found in the medial subiculum of adult Fmr1-KO mice [101].

Considering that VH in rodents and the corresponding anterior hippocampus in humans display increased susceptibility to epileptic/epileptiform activity compared with the dorsal hippocampus [56,62,64,66,68,77,78,79], the greatest impact of the increased excitability that accompanies FXS could be expected to occur specifically in the VH. Notably, relatively reduced GABAergic inhibition [51,52,53,54] has been suggested to crucially contribute to the characteristic tendency of the VH to epilepsy [50]. Therefore, upregulation of inhibition would prove beneficial, especially for VH-KO.

An interesting observation concerning FXS is that although a relatively high percentage of young individuals suffer hippocampus-involving epileptic seizures [3,30], epileptic discharges almost disappear in adults with FXS [3,33,34]. To our best knowledge, this notable age-dependent difference in susceptibility to epilepsy in FXS has not been previously explained.

The present findings that suggest an upregulation of GABAergic inhibition in VH of adult KO rats link the reduced vulnerability to epilepsy of adults with FXS, specifically with the ventral segment of the hippocampus, and provide a first mechanistic explanation for the reduced vulnerability to epileptic activity seen in the adult VH-KO. However, how this transformation occurs during development is not understood. Hypothetically, increased E-I balance and enhanced network excitability at early developmental stages may lead to compensatory adaptations that homeostatically attempt to restore normal activity in brain circuits [12,102,103,104]. Likewise, the deficiency in FMRP that leads to an initial primary deficit in the E-I balance may be followed by secondary changes occurring in the developing brain that act to adjust neuronal network activity to physiological levels. For instance, compensatory elevation in inhibition has been observed to follow an experimentally induced increase in E-I balance in cortical circuits, and it partially restores normal behavioral functions [105]. Additionally, compensatory changes have been suggested to occur from youth to adulthood in the hippocampus in the valproic acid-induced rat model of autism [106].

The present findings suggest that VH-KO is characterized by an upregulation of phasic but not tonic GABA_A_R-mediated transmission that significantly contributes to restraining epileptiform activity in this segment of the hippocampus. Furthermore, considering that fast/phasic GABAergic inhibition in the rat CA1 hippocampal area starts around the end of the first postnatal week [107,108] and reaches maturity levels by 30–35 postnatal days [109,110], it appears likely that the upregulation of GABAergic transmission in VH-KO might occur between the first and fifth postnatal week. It has been previously shown that the number of GABAergic neurons remains normal in the DH of KO mice [13]. Although it is not known whether a similar stability also occurs in the ventral segment of the KO hippocampus, it is more likely that a change in the functionality of GABAergic transmission could underlie the observed increased effectiveness of inhibition in VH-KO, as we indeed show in this study. For instance, increased excitability of GABAergic basket cell terminals due to the downregulation of Kv1.1 potassium channels has been recently reported to result in heightened GABAergic transmission in the cerebellum of Fmr1-KO mice [28]. Furthermore, upregulation of GABA_A_Rs has been previously reported to occur in cortical neurons to homeostatically compensate for an imbalance in excitability [111], as well as under conditions of increased anxiety [112], which is typically associated with FXS [2,3].

Therefore, we propose that the elevation in inhibition, specifically in VH of Fmr1-KO adult rats, may result from adaptational mechanisms that try to keep the function of the local network into the physiological range, thereby reducing the likelihood of epileptic activity in adults with FXS. Interestingly, GABAergic transmission can be increased in chronic temporal lobe epilepsy [113,114].

## 5. Conclusions

In conclusion, the hippocampus of adult FXS rats has increased excitability. Furthermore, the VH, the segment of the structure with inherently increased excitability, is characterized by a parallel enhancement of recurrent inhibition and an upregulation of α1GABA_A_Rs, but not α5GABA_A_Rs, that presumably keep the E-I ratio balanced, thereby preventing hyperexcitability and ensuring the physiological function of the ventral segment of the hippocampus.

## Data Availability

The data presented in this study are available on request from the corresponding author.

## References

[1] Kooy R.F., D’Hooge R., Reyniers E., Bakker C.E., Nagels G., De Boulle K., Storm K., Clincke G., De Deyn P.P., Oostra B.A. (1996). Transgenic mouse model for the fragile X syndrome. Am. J. Med. Genet..

[2] Hagerman R.J., Berry-Kravis E., Hazlett H.C., Bailey D.B., Moine H., Kooy R.F., Tassone F., Gantois I., Sonenberg N., Mandel J.L. (2017). Fragile X syndrome. Nat. Rev. Dis. Primers.

[3] Kidd S.A., Lachiewicz A., Barbouth D., Blitz R.K., Delahunty C., McBrien D., Visootsak J., Berry-Kravis E. (2014). Fragile X syndrome: A review of associated medical problems. Pediatrics.

[4] Kaufmann W.E., Kidd S.A., Andrews H.F., Budimirovic D.B., Esler A., Haas-Givler B., Stackhouse T., Riley C., Peacock G., Sherman S.L. (2017). Autism Spectrum Disorder in Fragile X Syndrome: Cooccurring Conditions and Current Treatment. Pediatrics.

[5] Bailey D.B., Mesibov G.B., Hatton D.D., Clark R.D., Roberts J.E., Mayhew L. (1998). Autistic behavior in young boys with fragile X syndrome. J. Autism Dev. Disord..

[6] Hagerman R.J., Jackson A.W., Levitas A., Rimland B., Braden M. (1986). An analysis of autism in fifty males with the fragile X syndrome. Am. J. Med. Genet..

[7] Verkerk A.J., Pieretti M., Sutcliffe J.S., Fu Y.H., Kuhl D.P., Pizzuti A., Reiner O., Richards S., Victoria M.F., Zhang F.P. (1991). Identification of a gene (FMR-1) containing a CGG repeat coincident with a breakpoint cluster region exhibiting length variation in fragile X syndrome. Cell.

[8] Bassell G.J., Warren S.T. (2008). Fragile X syndrome: Loss of local mRNA regulation alters synaptic development and function. Neuron.

[9] Richter J.D., Zhao X. (2021). The molecular biology of FMRP: New insights into fragile X syndrome. Nat. Rev. Neurosci..

[10] Booker S.A., Kind P.C. (2021). Mechanisms regulating input-output function and plasticity of neurons in the absence of FMRP. Brain Res. Bull..

[11] Sohal V.S., Rubenstein J.L.R. (2019). Excitation-inhibition balance as a framework for investigating mechanisms in neuropsychiatric disorders. Mol. Psychiatry.

[12] Nelson S.B., Valakh V. (2015). Excitatory/Inhibitory Balance and Circuit Homeostasis in Autism Spectrum Disorders. Neuron.

[13] Selby L., Zhang C., Sun Q.Q. (2007). Major defects in neocortical GABAergic inhibitory circuits in mice lacking the fragile X mental retardation protein. Neurosci. Lett..

[14] D’Hulst C., De Geest N., Reeve S.P., Van Dam D., De Deyn P.P., Hassan B.A., Kooy R.F. (2006). Decreased expression of the GABAA receptor in fragile X syndrome. Brain Res..

[15] D’Hulst C., Heulens I., Brouwer J.R., Willemsen R., De Geest N., Reeve S.P., De Deyn P.P., Hassan B.A., Kooy R.F. (2009). Expression of the GABAergic system in animal models for fragile X syndrome and fragile X associated tremor/ataxia syndrome (FXTAS). Brain Res..

[16] Sabanov V., Braat S., D’Andrea L., Willemsen R., Zeidler S., Rooms L., Bagni C., Kooy R.F., Balschun D. (2017). Impaired GABAergic inhibition in the hippocampus of Fmr1 knockout mice. Neuropharmacology.

[17] Adusei D.C., Pacey L.K., Chen D., Hampson D.R. (2010). Early developmental alterations in GABAergic protein expression in fragile X knockout mice. Neuropharmacology.

[18] El Idrissi A., Ding X.H., Scalia J., Trenkner E., Brown W.T., Dobkin C. (2005). Decreased GABA(A) receptor expression in the seizure-prone fragile X mouse. Neurosci. Lett..

[19] Davidovic L., Navratil V., Bonaccorso C.M., Catania M.V., Bardoni B., Dumas M.E. (2011). A metabolomic and systems biology perspective on the brain of the fragile X syndrome mouse model. Genome Res..

[20] Braat S., D’Hulst C., Heulens I., De Rubeis S., Mientjes E., Nelson D.L., Willemsen R., Bagni C., Van Dam D., De Deyn P.P. (2015). The GABAA receptor is an FMRP target with therapeutic potential in fragile X syndrome. Cell Cycle.

[21] Wahlstrom-Helgren S., Klyachko V.A. (2015). GABAB receptor-mediated feed-forward circuit dysfunction in the mouse model of fragile X syndrome. J. Physiol..

[22] Zhang N., Peng Z., Tong X., Lindemeyer A.K., Cetina Y., Huang C.S., Olsen R.W., Otis T.S., Houser C.R. (2017). Decreased surface expression of the δ subunit of the GABA(A) receptor contributes to reduced tonic inhibition in dentate granule cells in a mouse model of fragile X syndrome. Exp. Neurol..

[23] Paluszkiewicz S.M., Olmos-Serrano J.L., Corbin J.G., Huntsman M.M. (2011). Impaired inhibitory control of cortical synchronization in fragile X syndrome. J. Neurophysiol..

[24] Gibson J.R., Bartley A.F., Hays S.A., Huber K.M. (2008). Imbalance of neocortical excitation and inhibition and altered UP states reflect network hyperexcitability in the mouse model of fragile X syndrome. J. Neurophysiol..

[25] Morin-Parent F., Champigny C., Lacroix A., Corbin F., Lepage J.F. (2019). Hyperexcitability and impaired intracortical inhibition in patients with fragile-X syndrome. Transl. Psychiatry.

[26] Conde V., Palomar F.J., Lama M.J., Martínez R., Carrillo F., Pintado E., Mir P. (2013). Abnormal GABA-mediated and cerebellar inhibition in women with the fragile X premutation. J. Neurophysiol..

[27] Cea-Del Rio C.A., Nunez-Parra A., Freedman S.M., Kushner J.K., Alexander A.L., Restrepo D., Huntsman M.M. (2020). Disrupted inhibitory plasticity and homeostasis in Fragile X syndrome. Neurobiol. Dis..

[28] Yang Y.M., Arsenault J., Bah A., Krzeminski M., Fekete A., Chao O.Y., Pacey L.K., Wang A., Forman-Kay J., Hampson D.R. (2020). Identification of a molecular locus for normalizing dysregulated GABA release from interneurons in the Fragile X brain. Mol. Psychiatry.

[29] Liu X., Kumar V., Tsai N.P., Auerbach B.D. (2021). Hyperexcitability and Homeostasis in Fragile X Syndrome. Front. Mol. Neurosci..

[30] Berry-Kravis E. (2002). Epilepsy in fragile X syndrome. Dev. Med. Child Neurol..

[31] Incorpora G., Sorge G., Sorge A., Pavone L. (2002). Epilepsy in fragile X syndrome. Brain Dev..

[32] Kluger G., Böhm I., Laub M.C., Waldenmaier C. (1996). Epilepsy and fragile X gene mutations. Pediatr. Neurol..

[33] Berry-Kravis E., Filipink R.A., Frye R.E., Golla S., Morris S.M., Andrews H., Choo T.H., Kaufmann W.E. (2021). Seizures in Fragile X Syndrome: Associations and Longitudinal Analysis of a Large Clinic-Based Cohort. Front. Pediatr..

[34] Sabaratnam M., Vroegop P.G., Gangadharan S.K. (2001). Epilepsy and EEG findings in 18 males with fragile X syndrome. Seizure.

[35] Wisniewski K.E., French J.H., Fernando S., Brown W.T., Jenkins E.C., Friedman E., Hill A.L., Miezejeski C.M. (1985). Fragile X syndrome: Associated neurological abnormalities and developmental disabilities. Ann. Neurol..

[36] Anagnostou E., Taylor M.J. (2011). Review of neuroimaging in autism spectrum disorders: What have we learned and where we go from here. Mol. Autism.

[37] Varghese M., Keshav N., Jacot-Descombes S., Warda T., Wicinski B., Dickstein D.L., Harony-Nicolas H., De Rubeis S., Drapeau E., Buxbaum J.D. (2017). Autism spectrum disorder: Neuropathology and animal models. Acta Neuropathol..

[38] Fetit R., Hillary R.F., Price D.J., Lawrie S.M. (2021). The neuropathology of autism: A systematic review of post-mortem studies of autism and related disorders. Neurosci. Biobehav. Rev..

[39] Liu C., Liu J., Gong H., Liu T., Li X., Fan X. (2023). Implication of Hippocampal Neurogenesis in Autism Spectrum Disorder: Pathogenesis and Therapeutic Implications. Curr. Neuropharmacol..

[40] Banker S.M., Gu X., Schiller D., Foss-Feig J.H. (2021). Hippocampal contributions to social and cognitive deficits in autism spectrum disorder. Trends Neurosci..

[41] Ordemann G.J., Apgar C.J., Chitwood R.A., Brager D.H. (2021). Altered A-type potassium channel function impairs dendritic spike initiation and temporoammonic long-term potentiation in Fragile X syndrome. J. Neurosci..

[42] Strange B.A., Witter M.P., Lein E.S., Moser E.I. (2014). Functional organization of the hippocampal longitudinal axis. Nat. Rev. Neurosci..

[43] Bannerman D.M., Sprengel R., Sanderson D.J., McHugh S.B., Rawlins J.N., Monyer H., Seeburg P.H. (2014). Hippocampal synaptic plasticity, spatial memory and anxiety. Nat. Rev. Neurosci..

[44] Gulyaeva N.V. (2019). Functional Neurochemistry of the Ventral and Dorsal Hippocampus: Stress, Depression, Dementia and Remote Hippocampal Damage. Neurochem. Res..

[45] Bakoyiannis I., Ducourneau E.G., Parkes S.L., Ferreira G. (2023). Pathway specific interventions reveal the multiple roles of ventral hippocampus projections in cognitive functions. Rev. Neurosci..

[46] Risold P.Y., Swanson L.W. (1996). Structural evidence for functional domains in the rat hippocampus. Science.

[47] Van Groen T., Lopes da Silva F.H. (1985). Septotemporal distribution of entorhinal projections to the hippocampus in the cat: Electrophysiological evidence. J. Comp. Neurol..

[48] Pikkarainen M., Ronkko S., Savander V., Insausti R., Pitkanen A. (1999). Projections from the lateral, basal, and accessory basal nuclei of the amygdala to the hippocampal formation in rat. J. Comp. Neurol..

[49] van Strien N.M., Cappaert N.L., Witter M.P. (2009). The anatomy of memory: An interactive overview of the parahippocampal-hippocampal network. Nat. Rev. Neurosci..

[50] Papatheodoropoulos C. (2018). Electrophysiological evidence for long-axis intrinsic diversification of the hippocampus. Front. Biosci. (Landmark Ed.).

[51] Petrides T., Georgopoulos P., Kostopoulos G., Papatheodoropoulos C. (2007). The GABAA receptor-mediated recurrent inhibition in ventral compared with dorsal CA1 hippocampal region is weaker, decays faster and lasts less. Exp. Brain Res..

[52] Maggio N., Segal M. (2009). Differential corticosteroid modulation of inhibitory synaptic currents in the dorsal and ventral hippocampus. J. Neurosci. Off. J. Soc. Neurosci..

[53] Milior G., Castro M.A., Sciarria L.P., Garofalo S., Branchi I., Ragozzino D., Limatola C., Maggi L. (2016). Electrophysiological Properties of CA1 Pyramidal Neurons along the Longitudinal Axis of the Mouse Hippocampus. Sci. Rep..

[54] Papatheodoropoulos C., Asprodini E., Nikita I., Koutsona C., Kostopoulos G. (2002). Weaker synaptic inhibition in CA1 region of ventral compared to dorsal rat hippocampal slices. Brain Res..

[55] Netsyk O., Hammoud H., Korol S.V., Jin Z., Tafreshiha A.S., Birnir B. (2020). Tonic GABA-activated synaptic and extrasynaptic currents in dentate gyrus granule cells and CA3 pyramidal neurons along the mouse hippocampal dorsoventral axis. Hippocampus.

[56] Spencer D.D., Spencer S.S., Mattson R.H., Williamson P.D., Novelly R.A. (1984). Access to the posterior medial temporal lobe structures in the surgical treatment of temporal lobe epilepsy. Neurosurgery.

[57] Babb T.L., Brown W.J., Pretorius J., Davenport C., Lieb J.P., Crandall P.H. (1984). Temporal lobe volumetric cell densities in temporal lobe epilepsy. Epilepsia.

[58] Quigg M., Bertram E.H., Jackson T. (1997). Longitudinal distribution of hippocampal atrophy in mesial temporal lobe epilepsy. Epilepsy Res..

[59] Traub R.D., Jefferys J.G., Miles R. (1993). Analysis of the propagation of disinhibition-induced after-discharges along the guinea-pig hippocampal slice in vitro. J. Physiol..

[60] Derchansky M., Shahar E., Wennberg R.A., Samoilova M., Jahromi S.S., Abdelmalik P.A., Zhang L., Carlen P.L. (2004). Model of frequent, recurrent, and spontaneous seizures in the intact mouse hippocampus. Hippocampus.

[61] Gilbert M., Racine R.J., Smith G.K. (1985). Epileptiform burst responses in ventral vs dorsal hippocampal slices. Brain Res..

[62] Bragdon A.C., Taylor D.M., Wilson W.A. (1986). Potassium-induced epileptiform activity in area CA3 varies markedly along the septotemporal axis of the rat hippocampus. Brain Res..

[63] Lee P.H., Xie C.W., Lewis D.V., Wilson W.A., Mitchell C.L., Hong J.S. (1990). Opioid-induced epileptiform bursting in hippocampal slices: Higher susceptibility in ventral than dorsal hippocampus. J. Pharmacol. Exp. Ther..

[64] Papatheodoropoulos C., Moschovos C., Kostopoulos G. (2005). Greater contribution of N-methyl-D-aspartic acid receptors in ventral compared to dorsal hippocampal slices in the expression and long-term maintenance of epileptiform activity. Neuroscience.

[65] Papatheodoropoulos C. (2007). NMDA receptor-dependent high-frequency network oscillations (100-300 Hz) in rat hippocampal slices. Neurosci. Lett..

[66] Moschovos C., Kostopoulos G., Papatheodoropoulos C. (2012). Endogenous adenosine induces NMDA receptor-independent persistent epileptiform discharges in dorsal and ventral hippocampus via activation of A2 receptors. Epilepsy Res..

[67] Papatheodoropoulos C. (2015). Higher intrinsic network excitability in ventral compared with the dorsal hippocampus is controlled less effectively by GABAB receptors. BMC Neurosci..

[68] Mikroulis A.V., Psarropoulou C. (2012). Endogenous ACh effects on NMDA-induced interictal-like discharges along the septotemporal hippocampal axis of adult rats and their modulation by an early life generalized seizure. Epilepsia.

[69] Kouvaros S., Papatheodoropoulos C. (2016). Theta burst stimulation-induced LTP: Differences and similarities between the dorsal and ventral CA1 hippocampal synapses. Hippocampus.

[70] Papatheodoropoulos C., Kostopoulos G. (2000). Dorsal-ventral differentiation of short-term synaptic plasticity in rat CA1 hippocampal region. Neurosci. Lett..

[71] Maggio N., Segal M. (2007). Unique regulation of long term potentiation in the rat ventral hippocampus. Hippocampus.

[72] Kenney J., Manahan-Vaughan D. (2013). NMDA receptor-dependent synaptic plasticity in dorsal and intermediate hippocampus exhibits distinct frequency-dependent profiles. Neuropharmacology.

[73] Papaleonidopoulos V., Papatheodoropoulos C. (2018). β-adrenergic receptors reduce the threshold for induction and stabilization of LTP and enhance its magnitude via multiple mechanisms in the ventral but not the dorsal hippocampus. Neurobiol. Learn. Mem..

[74] Kouvaros S., Papatheodoropoulos C. (2016). Major dorsoventral differences in the modulation of the local CA1 hippocampal network by NMDA, mGlu5, adenosine A2A and cannabinoid CB1 receptors. Neuroscience.

[75] Sieghart W., Sperk G. (2002). Subunit composition, distribution and function of GABA(A) receptor subtypes. Curr. Top. Med. Chem..

[76] Brunig I., Scotti E., Sidler C., Fritschy J.M. (2002). Intact sorting, targeting, and clustering of γ-aminobutyric acid A receptor subtypes in hippocampal neurons in vitro. J. Comp. Neurol..

[77] Akaike K., Tanaka S., Tojo H., Fukumoto S., Imamura S., Takigawa M. (2001). Kainic acid-induced dorsal and ventral hippocampal seizures in rats. Brain Res..

[78] Greco B., Prevost J., Gioanni Y. (1994). Intracerebral microinjections of dermorphin: Search for the epileptic induction thresholds. Neuroreport.

[79] Haussler U., Bielefeld L., Froriep U.P., Wolfart J., Haas C.A. (2012). Septotemporal position in the hippocampal formation determines epileptic and neurogenic activity in temporal lobe epilepsy. Cereb. Cortex.

[80] Bai D., Zhu G., Pennefather P., Jackson M.F., MacDonald J.F., Orser B.A. (2001). Distinct functional and pharmacological properties of tonic and quantal inhibitory postsynaptic currents mediated by γ-aminobutyric acid(A) receptors in hippocampal neurons. Mol. Pharmacol..

[81] Bieda M.C., MacIver M.B. (2004). Major role for tonic GABAA conductances in anesthetic suppression of intrinsic neuronal excitability. J. Neurophysiol..

[82] Kullmann D.M., Ruiz A., Rusakov D.M., Scott R., Semyanov A., Walker M.C. (2005). Presynaptic, extrasynaptic and axonal GABAA receptors in the CNS: Where and why?. Prog. Biophys. Mol. Biol..

[83] Caraiscos V.B., Elliott E.M., You-Ten K.E., Cheng V.Y., Belelli D., Newell J.G., Jackson M.F., Lambert J.J., Rosahl T.W., Wafford K.A. (2004). Tonic inhibition in mouse hippocampal CA1 pyramidal neurons is mediated by α5 subunit-containing γ-aminobutyric acid type A receptors. Proc. Natl. Acad. Sci. USA.

[84] Prenosil G.A., Schneider Gasser E.M., Rudolph U., Keist R., Fritschy J.M., Vogt K.E. (2006). Specific subtypes of GABAA receptors mediate phasic and tonic forms of inhibition in hippocampal pyramidal neurons. J. Neurophysiol..

[85] Sur C., Quirk K., Dewar D., Atack J., McKernan R. (1998). Rat and human hippocampal alpha5 subunit-containing gamma-aminobutyric AcidA receptors have alpha5 beta3 gamma2 pharmacological characteristics. Mol. Pharmacol..

[86] Glykys J., Mann E.O., Mody I. (2008). Which GABA(A) receptor subunits are necessary for tonic inhibition in the hippocampus?. J. Neurosci. Off. J. Soc. Neurosci..

[87] Pandit S., Lee G.S., Park J.B. (2017). Developmental changes in GABA(A) tonic inhibition are compromised by multiple mechanisms in preadolescent dentate gyrus granule cells. Korean J. Physiol. Pharmacol. Off. J. Korean Physiol. Soc. Korean Soc. Pharmacol..

[88] Chuang S.C., Zhao W., Bauchwitz R., Yan Q., Bianchi R., Wong R.K. (2005). Prolonged epileptiform discharges induced by altered group I metabotropic glutamate receptor-mediated synaptic responses in hippocampal slices of a fragile X mouse model. J. Neurosci. Off. J. Soc. Neurosci..

[89] Gross C., Yao X., Pong D.L., Jeromin A., Bassell G.J. (2011). Fragile X mental retardation protein regulates protein expression and mRNA translation of the potassium channel Kv4.2. J. Neurosci. Off. J. Soc. Neurosci..

[90] Kalmbach B.E., Johnston D., Brager D.H. (2015). Cell-Type Specific Channelopathies in the Prefrontal Cortex of the fmr1-/y Mouse Model of Fragile X Syndrome. eNeuro.

[91] Luque M.A., Beltran-Matas P., Marin M.C., Torres B., Herrero L. (2017). Excitability is increased in hippocampal CA1 pyramidal cells of Fmr1 knockout mice. PLoS ONE.

[92] Deng P.Y., Carlin D., Oh Y.M., Myrick L.K., Warren S.T., Cavalli V., Klyachko V.A. (2019). Voltage-Independent SK-Channel Dysfunction Causes Neuronal Hyperexcitability in the Hippocampus of Fmr1 Knock-Out Mice. J. Neurosci. Off. J. Soc. Neurosci..

[93] Booker S.A., Simões de Oliveira L., Anstey N.J., Kozic Z., Dando O.R., Jackson A.D., Baxter P.S., Isom L.L., Sherman D.L., Hardingham G.E. (2020). Input-Output Relationship of CA1 Pyramidal Neurons Reveals Intact Homeostatic Mechanisms in a Mouse Model of Fragile X Syndrome. Cell Rep..

[94] Asiminas A., Booker S.A., Dando O.R., Kozic Z., Arkell D., Inkpen F.H., Sumera A., Akyel I., Kind P.C., Wood E.R. (2022). Experience-dependent changes in hippocampal spatial activity and hippocampal circuit function are disrupted in a rat model of Fragile X Syndrome. Mol. Autism.

[95] Paluszkiewicz S.M., Martin B.S., Huntsman M.M. (2011). Fragile X syndrome: The GABAergic system and circuit dysfunction. Dev. Neurosci..

[96] Nomura T. (2021). Interneuron Dysfunction and Inhibitory Deficits in Autism and Fragile X Syndrome. Cells.

[97] Deng P.Y., Kumar A., Cavalli V., Klyachko V.A. (2022). FMRP regulates GABA(A) receptor channel activity to control signal integration in hippocampal granule cells. Cell Rep..

[98] Kang J.Y., Chadchankar J., Vien T.N., Mighdoll M.I., Hyde T.M., Mather R.J., Deeb T.Z., Pangalos M.N., Brandon N.J., Dunlop J. (2017). Deficits in the activity of presynaptic γ-aminobutyric acid type B receptors contribute to altered neuronal excitability in fragile X syndrome. J. Biol. Chem..

[99] Hong A., Zhang A., Ke Y., El Idrissi A., Shen C.H. (2012). Downregulation of GABA(A) β subunits is transcriptionally controlled by Fmr1p. J. Mol. Neurosci. MN.

[100] Vicini S., Ferguson C., Prybylowski K., Kralic J., Morrow A.L., Homanics G.E. (2001). GABA(A) receptor alpha1 subunit deletion prevents developmental changes of inhibitory synaptic currents in cerebellar neurons. J. Neurosci. Off. J. Soc. Neurosci..

[101] Curia G., Papouin T., Séguéla P., Avoli M. (2009). Downregulation of tonic GABAergic inhibition in a mouse model of fragile X syndrome. Cereb. Cortex.

[102] Turrigiano G.G., Nelson S.B. (2004). Homeostatic plasticity in the developing nervous system. Nat. Rev. Neurosci..

[103] Howard M.A., Rubenstein J.L., Baraban S.C. (2014). Bidirectional homeostatic plasticity induced by interneuron cell death and transplantation in vivo. Proc. Natl. Acad. Sci. USA.

[104] Briggs S.W., Galanopoulou A.S. (2011). Altered GABA signaling in early life epilepsies. Neural Plast..

[105] Yizhar O., Fenno L.E., Prigge M., Schneider F., Davidson T.J., O’Shea D.J., Sohal V.S., Goshen I., Finkelstein J., Paz J.T. (2011). Neocortical excitation/inhibition balance in information processing and social dysfunction. Nature.

[106] Bódi V., Májer T., Kelemen V., Világi I., Szűcs A., Varró P. (2022). Alterations of the Hippocampal Networks in Valproic Acid-Induced Rat Autism Model. Front. Neural Circuits.

[107] Harris K.M., Teyler T.J. (1983). Evidence for late development of inhibition in area CA1 of the rat hippocampus. Brain Res..

[108] Swann J.W., Brady R.J., Martin D.L. (1989). Postnatal development of GABA-mediated synaptic inhibition in rat hippocampus. Neuroscience.

[109] Hutcheon B., Fritschy J.M., Poulter M.O. (2004). Organization of GABA receptor alpha-subunit clustering in the developing rat neocortex and hippocampus. Eur. J. Neurosci..

[110] Banks M.I., Hardie J.B., Pearce R.A. (2002). Development of GABA(A) receptor-mediated inhibitory postsynaptic currents in hippocampus. J. Neurophysiol..

[111] Chen X., Shu S., Schwartz L.C., Sun C., Kapur J., Bayliss D.A. (2010). Homeostatic regulation of synaptic excitability: Tonic GABA(A) receptor currents replace I(h) in cortical pyramidal neurons of HCN1 knock-out mice. J. Neurosci. Off. J. Soc. Neurosci..

[112] Chen Y.W., Actor-Engel H., Aoki C. (2018). α4-GABA(A) receptors of hippocampal pyramidal neurons are associated with resilience against activity-based anorexia for adolescent female mice but not for males. Mol. Cell. Neurosci..

[113] Esclapez M., Houser C.R. (1999). Up-regulation of GAD65 and GAD67 in remaining hippocampal GABA neurons in a model of temporal lobe epilepsy. J. Comp. Neurol..

[114] Bernard C., Cossart R., Hirsch J.C., Esclapez M., Ben-Ari Y. (2000). What is GABAergic inhibition? How is it modified in epilepsy?. Epilepsia.

